# Diazoxide Choline Extended-release Tablets in Prader-Willi Syndrome: A Randomized, Double-blind, Withdrawal Period Study

**DOI:** 10.1210/clinem/dgaf661

**Published:** 2026-01-02

**Authors:** Jennifer L Miller, Nicola Bridges, Eric I Felner, Parisa Salehi, Jack A Yanovski, David A Stevenson, Jorge Mejia-Corletto, Mohamad G Shaikh, M Jennifer Abuzzahab, Amy Fleischman, Virginia Kimonis, Ashley H Shoemaker, Anthony Holland, Lynne M Bird, Kathryn S Obrynba, Melissa Lah, Elizabeth Littlejohn, Katerina Harwood, Heidi Shea, David Viskochil, Patricia Hirano, Kristen Yen, Shaila Ballal, Michael Huang, Neil M Cowen, Anish Bhatnagar, Evelien Gevers

**Affiliations:** Department of Pediatric Endocrinology, University of Florida College of Medicine, Gainesville, FL 32608, USA; Department of Endocrinology, Chelsea and Westminster Hospital, London SW10 9NH, UK; Department of Pediatrics, Emory University School of Medicine, Atlanta, GA 30322, USA; Endocrinology, Seattle Children's Hospital, Seattle, WA 98105, USA; US Eunice Kennedy Shriver National Institute of Child Health and Human Development, National Institutes of Health, Bethesda, MD 20892, USA; Department of Clinical Genetics, Stanford University, Palo Alto, CA 94305, USA; Department of Pediatric Endocrinology, NYU Langone Health, Mineola, NY 11501, USA; Department of Endocrinology, Royal Hospital for Children, Glasgow G51 4TF, UK; Department of Endocrinology, Children's Minnesota, St. Paul, MN 55102, USA; Division of Endocrinology, Boston Children's Hospital, Boston, MA 02115, USA; Division of Endocrinology, University of California, Irvine, and Children’s Hospital of Orange County, Orange, CA 92868, USA; Department of Pediatrics, Vanderbilt University Medical Center, Nashville, TN 37212, USA; Department of Psychiatry, Cambridgeshire & Peterborough NHS Foundation Trust, Fulbourn Hospital, Cambridge CB21 5EF, UK; Department of Genetics, University of California, San Diego/Rady Childrens Hospital, San Diego, CA 92123, USA; Endocrinology, Nationwide Children's Hospital, Columbus, OH 43205, USA; Department of Pediatrics, Indiana University School of Medicine, Indianapolis, IN 46202, USA; Department of Pediatric Endocrinology, University of Michigan Health—Sparrow Clinical Research Institute, Lansing, MI 48912, USA; Department of Pediatric Endocrinology, St. Joseph's University Medical Center, Paterson, NJ 07503, USA; Endocrinology, Research Institute of Dallas, Dallas, TX 75231, USA; Department of Pediatrics, University of Utah, Salt Lake City, UT 84108, USA; Soleno Therapeutics, Redwood City, CA 94065, USA; Soleno Therapeutics, Redwood City, CA 94065, USA; Soleno Therapeutics, Redwood City, CA 94065, USA; Soleno Therapeutics, Redwood City, CA 94065, USA; Soleno Therapeutics, Redwood City, CA 94065, USA; Soleno Therapeutics, Redwood City, CA 94065, USA; Endocrinology, Queen Mary University London, London E1 4NS, UK; Endocrinology, Barts Health NHS Trust-Royal London Children's Hospital, London E1 1FR, UK

**Keywords:** diazoxide choline extended-release tablets, Prader-Willi syndrome, hyperphagia, randomized withdrawal period study

## Abstract

**Context:**

The hallmark condition of Prader-Willi syndrome, a rare, genetic neurobehavioral/metabolic disorder, is life-threatening hyperphagia.

**Objective:**

We assessed the efficacy and safety of diazoxide choline extended-release (DCCR) tablets for the treatment of hyperphagia in adults and children four years of age and older with Prader-Willi syndrome.

**Methods:**

We conducted a 16-week, randomized withdrawal study in children and adults with Prader-Willi syndrome and hyperphagia. Participants who previously completed randomized (13-week DCCR or placebo) and open-label (2.5-4.5 years DCCR) studies were randomized 1:1 to receive once-daily DCCR or placebo. The primary endpoint was Hyperphagia Questionnaire for Clinical Trials (HQ-CT) total score change from baseline to 16 weeks. Secondary endpoints included Clinical Global Impression of Severity (CGI-S) and Improvement (CGI-I); exploratory endpoints included weight and body mass index (BMI) z-score.

**Results:**

Seventy-seven participants were randomized (DCCR:38; placebo:39). Statistically significant increases in HQ-CT from baseline to week 16 were observed with placebo vs DCCR [least square (LS) mean (standard error) change 7.6 (1.09)] with placebo and 2.6 (1.12) with DCCR; *P* = .0022. CGI scores favored DCCR but were not significantly changed. Consistent with the hyperphagia response, the placebo cohort gained more weight and increased their BMI z-score more than the DCCR cohort [LS mean weight difference (95% confidence interval) −1.6 kg (−3.1, −0.1)]; LS mean z-score difference was −0.09 (−0.17, −0.01). Adverse events were similar with both treatments, with no serious adverse events in the DCCR arm.

**Conclusion:**

Continued DCCR treatment was superior to placebo for hyperphagia. DCCR appears to offer meaningful therapeutic benefits for people with Prader-Willi syndrome.

Prader-Willi syndrome (PWS) is a rare, complex genetic neurobehavioral/metabolic disorder with an estimated birth incidence of 1:15 000 to 1:20 000 (1, 2). Clinical manifestations of PWS include hypotonia and feeding difficulties in infancy (3). PWS is also associated with intellectual disability; low muscle mass; growth and sex hormone deficiency; and behavioral problems, including aggression, anxiety, and compulsivity. Accumulation of excess body fat often begins at an earlier age, before onset of hyperphagia. Hyperphagia, a life-threatening hallmark of the syndrome, presenting as food obsession, aggressive food seeking, and lack of satiety, occurs at a median age of 8 years but can begin as early as 3 to 4 years old. There is elevated risk of early mortality in PWS, with a median age of death between 21 and 29 years (4, 5). Hyperphagia is associated with risk of death due to gastrointestinal perforation, choking, and accidents during food seeking and may account for one-third of all reported deaths and one-half of deaths in childhood (6). Kayadjanian et al examined the contribution of hyperphagia to caregiver burden across the lifespan using the Hyperphagia Questionnaire for Clinical Trials (HQ-CT) and the Zarit Burden Interview. The authors found a strong correlation between the Zarit Burden Interview and HQ-CT in individuals with PWS older than 4 years (7). Similarly, siblings of people with PWS nearly all have at least 1 symptom of posttraumatic stress disorder, and nearly 30% exhibit clinically relevant symptoms of posttraumatic stress disorder (8).

Diazoxide choline is a potent activator of the adenosine triphosphate-sensitive potassium channel and is capable of crossing the blood-brain barrier (9). Diazoxide choline extended-release (DCCR) facilitates once-per-day dosing with stable and predictable intraday plasma concentrations (10, 11). In PWS, the loss of SNORD116 results in elevations in the synthesis and secretion of neuropeptide Y (NPY), agouti related-protein (AgRP), and likely gamma aminobutyric acid (GABA) by NPY/AgRP/GABA neurons. The secretion of these potent endogenous orexigenic neuropeptides and neurotransmitters is further dysregulated by central resistance to the action of leptin and insulin (12, 13). Activation of the adenosine triphosphate-sensitive potassium channel in NPY/AgRP/GABA neurons in the hypothalamus hyperpolarizes the plasma membrane potential, preventing fusion of secretory vesicles with the plasma membrane, reducing secretion of NPY, AgRP, and GABA, potent endogenous orexigenic neuropeptides and neurotransmitters, potentially reducing hyperphagia (14, 15).

DESTINY PWS was a randomized, double-blind, placebo-controlled, 13-week, phase 3 study comparing DCCR with placebo in participants with genetically confirmed PWS age 4 years and older with hyperphagia (10). Participants were enrolled at 29 sites in the United States and United Kingdom. No significant improvements in hyperphagia were noted between DCCR vs placebo arms at 13 weeks, which may have been due to the limited amount of time spent at the target dose (7 weeks) and the onset of the COVID-19 pandemic (10). The results of 52 weeks of DCCR administration in the long-term, open-label, follow-up study to DESTINY PWS showed significant improvements in hyperphagia and several other behavioral and metabolic endpoints as compared to baseline (11) and to a nonintervention external control group (16).

We conducted a randomized double-blind, placebo-controlled, withdrawal period [randomized withdrawal period (RWP)] study of participants with PWS in the open-label study to assess the effect of continued treatment with DCCR compared to the effect of withdrawal of DCCR and initiation of placebo treatment to determine the long-term efficacy of DCCR for hyperphagia.

## Methods

### Study Design

The DESTINY PWS-RWP was a multicenter 16-week randomized, placebo-controlled, withdrawal study in people with genetically confirmed PWS with hyperphagia who completed a double-blind study (DESTINY PWS) and its long-term, open-label follow-on study of DCCR (10, 11). Eligible participants who consented to the study were randomized 1:1 to receive DCCR or placebo. Randomization was stratified by HQ-CT total score at the RWP baseline (stratum 1: randomized withdrawal baseline HQ-CT total score <13; stratum 2: randomized withdrawal baseline HQ-CT total score 13-36).

The trial was conducted in accordance with the International Council for Harmonisation guidelines for Good Clinical Practice; the ethical principles of the Declaration of Helsinki; and relevant applicable laws, regulations, and/or legislation. The trial was approved for each center by an institutional review board or independent ethics committee. Participants or their parents/guardians provided written informed consent and, as appropriate, assent prior to being enrolled in the study. Confidentiality agreements were in place between the sponsor and site investigators. A data safety monitoring board provided safety oversight for the study. All authors vouch the data are complete and accurate, for the fidelity of the trial to the protocol, and for the full reporting of adverse events (AEs).

### Participants

To be randomized, participants taking open-label DCCR had to provide voluntary consent and assent (as appropriate) to the study procedures and complete the protocol-defined procedures after the end of the open-label phase.

### Randomization and Masking

To reduce the potential for bias in the RWP, randomized assignment to DCCR or placebo was double-blinded. The participants, caregivers, study-site personnel, and the sponsor and its representatives involved in the direct conduct and/or management of the study were not informed of the assigned treatment and remained blinded to the randomization codes until after the study and analysis were completed. Treatment was blinded for any data reviews prior to database lock and unblinding. Study medication, including its packaging and labeling, was blinded. Placebo tablets matching the size, shape, and color of the respective DCCR tablet strengths were used.

### Procedures

DCCR or placebo was administered once daily. Dosing within the study was weight based and generally targeted a dose between 3.3 and 5.8 mg/kg of diazoxide choline. Based on their weight, individuals were assigned to 1 of 5 weight bands at the start of DESTINY PWS, at which time participants were excluded if they weighed <20 kg or ≥135 kg. Titration steps and target dose varied by weight band. Thereafter, dose adjustments could be made for either safety or efficacy. The visits at baseline and at 16 weeks were in clinic while the 3 other visits at 4, 8, and 12 weeks were telemedicine visits.

A full physical examination, including the measurement of weight, height, waist circumference, and vital signs were completed at baseline and at week 16.

The same caregiver of each participant completed the HQ-CT at each visit with guidance from trained site personnel. All site staff involved in the oversight of completion of these questionnaires received training on how to properly administer the HQ-CT consistently across participants and visits and to not provide any leading or suggestive responses to questions the caregiver may raise. During the study, the participant and caregiver did not have access to the scoring of the questionnaires or to responses from previously completed questionnaires that would influence their rating at a visit. In order to facilitate identification of changes in PWS, investigators and study site personnel had access to HQ-CT responses (responses to individual questions and total scores) completed by the caregiver.

The caregiver also completed the Prader-Willi Syndrome Profile (PWSP) and Food Safe Zone (FSZ) questionnaires and the Caregiver Global Impression Scales at each visit. The clinician completed the Clinical Global Impression scales at each visit.

Participants/caregivers were instructed to contact the study site in the event they experienced any AEs. At the discretion of the investigator, the participant may have been asked to come to the study site for an unscheduled visit or may have been referred to a local clinic or hospital for laboratory measurements, electrocardiograms, or other assessments as clinically indicated. AEs were coded using the Medical Dictionary for Regulatory Activities, Version 25.1.

### Outcomes

The primary endpoint of the RWP was change in HQ-CT total score from baseline to 16 weeks. The HQ-CT is a 9-item, caregiver-completed questionnaire assessing food-related behaviors associated with hyperphagia, with increases in HQ-CT representing worsening of hyperphagia (minimum score 0, maximum score 36). The HQ-CT has been validated to assess changes in hyperphagia in clinical trials in PWS (17). Secondary endpoints included the Clinical Global Impression of Severity (CGI-S) and the Clinical Global Impression of Improvement (CGI-I). Exploratory endpoints included weight; body mass index (BMI) z-score; the PWSP Questionnaire, which assesses the behavioral phenotype of PWS in 6 domains (aggressive behaviors, compulsivity, rigidity/irritability, anxiety, disordered thinking, and depression) (18); Caregiver Global Impression of Change (Caregiver GI-C) and of Severity (Caregiver GI-S); and the FSZ, assessing environmental controls on access to food in 5 domains. All endpoints except CGI-I (which is assessed as change from baseline) were assessed at baseline. Safety parameters included treatment-emergent AEs, routine laboratory tests, glycosylated hemoglobin A1c, and fasting glucose.

### Statistical Analysis

The change from baseline in HQ-CT total score was analyzed using a mixed model for repeated measures (MMRM) with fixed effects for treatment, visit, and treatment by visit interaction and using baseline HQ-CT total score as a continuous covariate. Least square (LS) mean differences (DCCR vs placebo) were obtained from the MMRM at each visit, with the comparison at week 16 being primary. CGI-S was analyzed similarly but adjusting for the baseline CGI-S score instead of the HQ-CT score. CGI-I was analyzed using a logistic regression proportional odds model adjusted for baseline HQ-CT strata and treatment. Domains of the PWSP were analyzed using MMRM. Weight and BMI z-score were analyzed using analysis of covariance. All comparisons were for superiority. If the change from baseline in HQ-CT total score was statistically significant at an α level of .05 (favoring DCCR vs placebo), then testing proceeded to CGI-S, CGI-I, and PWSP domain scores in order, as long as each test was statistically significant. There was no adjustment for multiplicity. Significance reported was to be nominal for the remaining exploratory endpoints.

An estimate of the power to be realized with this fixed sample size for the primary endpoint was made assuming the SD of change in HQ-CT total score is 7.0 (from DESTINY PWS) and a sample size of ≥74 participants would have at least 85% power to detect a statistically significant difference between DCCR and placebo if the underlying difference in change scores were 5.0 points.

### Role of the Funding Source

The sponsor designed the study with input from clinicians, patient advocacy organizations, and caregivers. The sites had a role in data collection, while an independent statistical contract research organization had a role in data analysis. Investigators and the sponsor had a role in data interpretation and writing of the report. Sponsor authors collaborated with academic authors in the development of the manuscript.

## Results

### Participants

**Figure 1.**
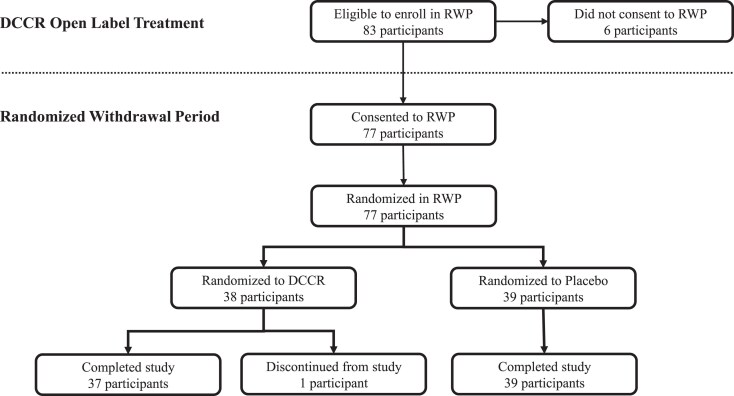
Consort diagram. Six participants who were eligible for enrollment in RWP declined consent. All who consented to the study were randomized. One participant discontinued from the study associated with a withdrawal of consent unrelated to safety. Abbreviations: DCCR, diazoxide choline extended-release; RWP, randomized withdrawal period.

Seventy-seven of 83 (92.8%) eligible participants consented to enroll in the DESTINY PWS-RWP study. The average reduction in HQ-CT total score from the initiation of DCCR administration (mean DESTINY PWS baseline HQ-CT total score was 22.6 points) to the baseline visit in the RWP was nearly 13 points. A total of 38 participants were randomized to continue DCCR, and 39 were randomized to receive placebo (Fig. 1). Seventy-six participants completed the study (Fig. 1). One DCCR participant was discontinued because the caregiver withdrew consent and not for safety reasons. Baseline and demographic characteristics are provided in Table 1. The baseline characteristics were compared between the cohorts. The only parameter for which there was a significant difference between the cohorts was weight, with participants randomized to DCCR being heavier; however, BMI was not significantly different. Otherwise, the arms were comparable, especially for HQ-CT total score.

**Table 1.** Demographics and baseline characteristics for DESTINY PWS-RWP

Parameter	DCCR (n = 38)	Placebo (n = 39)	*P*-value	All participants (n = 77)
Age (years), mean (SD)	15.6 (4.69)	14.2 (4.32)	.2025	14.9 (4.52)
Race, n (%)			.6330	
White	32 (84.2)	34 (87.2)		66 (85.7)
Black or African American	2 (5.3)	3 (7.7)		5 (6.5)
Multiple	4 (10.5)	2 (5.1)		6 (7.8)
Ethnicity, n (%)			.3901	
Hispanic or Latino	4 (10.5)	2 (5.1)		6 (7.8)
Not Hispanic or Latino	33 (86.8)	37 (94.9)		70 (90.9)
Not reported	1 (2.6)	0		1 (1.3)
Sex, n (%)			.1393	
Male	20 (52.6)	14 (35.9)		34 (44.2)
Female	18 (47.4)	25 (64.1)		43 (55.8)
Height (cm), mean (SD)	159.2 (15.53)	155.5 (13.50)	.2623	157.3 (14.56)
Weight (kg), mean (SD)	73.71 (29.31)	61.65 (16.59)	.0308	67.60 (24.34)
BMI z-score, mean (SD)	1.34 (1.125)	1.18 (0.894)	.5095	1.26 (1.060)
PWS genetic subtype, n (%)			.1267	
Deletion	22 (57.9)	29 (74.4)		51 (66.2)
Nondeletion	16 (42.1)	10 (25.6)		26 (33.8)
Country, n (%)			.4195	
United Kingdom	6 (15.8)	9 (23.1)		15 (19.5)
United States	32 (84.2)	30 (76.9)		62 (80.5)
HQ-CT total score, mean (SD)	9.0 (6.33)	8.1 (5.11)	.4604	8.5 (5.73)

Abbreviations: BMI, body mass index; DCCR, diazoxide choline extended-release; HQ-CT, Hyperphagia Questionnaire for Clinical Trials; PWS, Prader-Willi syndrome; RWP, randomized withdrawal period.

### Efficacy

**Figure 2.**
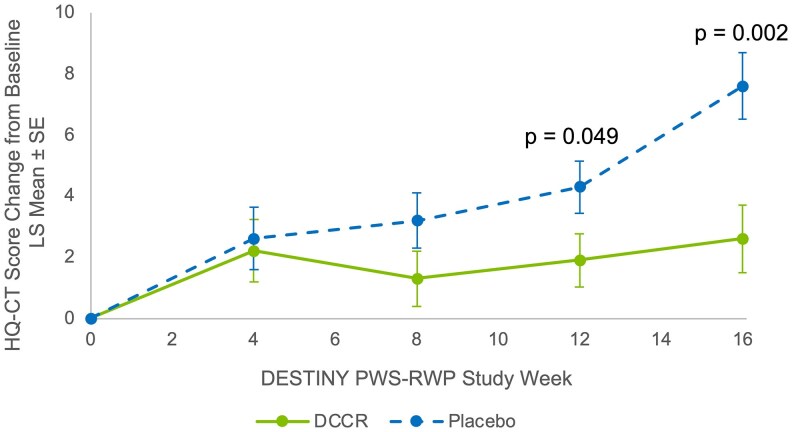
HQ-CT score change from baseline by study week and arm. HQ-CT least-square mean change from baseline (±SE). The HQ-CT was completed by caregivers of participants with Prader-Willi syndrome who were randomized to continue DCCR (shown in green solid line) or switched to placebo (shown in blue dashed line) during the RWP. The arms began to separate starting after week 4, with progressive separation of the arms from week 8 to week 16 with the difference first reaching significance at week 12. Abbreviations: DCCR, diazoxide choline extended-release; HQ-CT, Hyperphagia Questionnaire for Clinical Trials; RWP, randomized withdrawal period.

HQ-CT total score progressively worsened in participants randomized to placebo (Fig. 2), with a significant difference between the arms occurring by week 12. There was a statistically significant increase in HQ-CT from baseline at week 16 in the placebo arm compared to the DCCR arm (Fig. 2 and Table 2; LS mean [SE] change: 7.6 [1.09] in the placebo arm vs 2.6 [1.12] in the DCCR arm; *P* = .0022]. The results were not driven by a single question. There was a greater increase in score in the placebo cohort compared to the DCCR cohort for every question in the HQ-CT. There was nearly a 1-point change on 7 of the 9 questions (Fig. S1) (19). The results were also not driven by the highest enrolling site (Table S1) (19). Results were generally consistent across randomization strata and key subgroups that could affect PWS (Fig. S2) (19).

**Table 2.** DESTINY PWS-RWP efficacy results

	DCCR (n = 38)	Placebo (n = 39)	DCCR vs placebo
	Change from RWP baseline	Change from RWP baseline	Change from RWP baseline
Primary endpoint			
HQ-CT*^a^* LS, mean (SE)	2.6 (1.12)	7.6 (1.09)	−5.0 (1.57) *P* = .0022
Secondary endpoints			
CGI-S, LS mean (SE) or LS mean difference (CI)	0.9 (0.19)	1.4 (0.18)	−0.4 (−0.9, 0.1)
CGI-I, odds ratio (CI)			2.09 (0.89, 4.93)
Exploratory endpoints			
PWSP domain, LS mean (SE) or LS mean difference (CI)			
Aggressive behaviors	0.9 (0.66)	1.9 (0.66)	−1.0 (−2.8, 0.8)
Compulsivity	0.3 (0.61)	1.1 (0.61)	−0.8 (−2.4, 0.8)
Rigidity/irritability	0.3 (0.74)	1.8 (0.73)	−1.5 (−3.4, 0.5)
Anxiety	0.3 (0.61)	1.5 (0.62)	−1.2 (−2.8, 0.4)
Disordered thinking	−0.1 (0.30)	0.3 (0.30)	−0.5 (−1.3, 0.3)
Depression	0.2 (0.34)	0.9 (0.34)	−0.7 (−1.6, 0.2)
Caregiver GI-S, LS mean (SE) or LS mean difference (CI)	0.3 (0.13)	0.6 (0.12)	−0.3 (−0.6, 0.0)
Caregiver GI-C, LS mean (SE) or LS mean difference (CI)	4.1 (0.38)	4.9 (0.35)	−0.7 (−1.7, 0.2)
BMI, z-score, LS mean (SE) or LS mean difference (CI)	−0.02 (0.031)	0.07 (0.031)	−0.09 (−0.17, −0.01)
Food Safe Zone domain, LS mean (SE) or LS mean difference (CI)			
Restrict food access	0.2 (0.43)	1.3 (0.41)	−1.1 (−2.3, 0.0)
Check for food	0.4 (0.55)	1.7 (0.52)	−1.3 (−2.8, 0.1)
Food supervision with others	0.2 (0.32)	0.7 (0.31)	−0.5 (−0.3, 0.4)
Food supervision at home	−0.1 (0.30)	0.5 (0.29)	−0.7 (−1.5, 0.1)
Avoid unpredictable food situations	0.3 (0.33)	0.6 (0.32)	−0.3 (−1.2, 0.6)

Abbreviations: BMI, body mass index; Caregiver GI-C, Caregiver Global Impression of Change; Caregiver GI-S, Caregiver Global Impression of Severity; CGI-I, Clinical Global Impression of Improvement; CGI-S, Clinical Global Impression of Severity; CI, confidence interval; DCCR, diazoxide choline extended-release; HQ-CT, Hyperphagia Questionnaire for Clinical Trials; LS mean, least square mean; PWSP, Prader-Willi Syndrome Profile; RWP, randomized withdrawal period; SE, standard error.

^*a*^Increases in score reflecting worsening.

A set of analyses was run to generate supportive evidence for the interpretation of the HQ-CT total score using data collected in the RWP. Anchor-based methods were primarily used to determine the clinically meaningful within-patient worsening in the HQ-CT total score. Caregiver Global Impression of Severity (Caregiver GI-S, primary anchor), Caregiver GI-C, CGI-I, CGI-S, and Caregiver GI-S of Food-Related Behaviors were identified as potential anchor variables for the analyses. Anchor-based methods concluded that the meaningful within-patient worsening in HQ-CT total score ranged between 3 to 7 points, 4 to 7 points, or 5 to 7 points depending on the anchor variable used. Distribution-based estimates were between 2.5 and 3 points. These analyses identified an increase in the HQ-CT total score of 5 points as a reference estimate for the clinically meaningful within-patient worsening, with an associated range of 3 to 7 points for sensitivity analyses.

The CGI-S change from baseline comparison between arms was not significant (LS mean difference [95% confidence interval (CI)] −0.4 [−0.9, 0.1]; Fig. S3) (19). The CGI-I comparison between the arms was not significant (odds ratio for placebo vs DCCR [95% CI] 2.09 [0.89, 4.93]; Fig. S4) (19). While there was less worsening for all 6 domains of the PWSP in the DCCR arm than in the placebo arm, no domain reached statistical significance. Since participants in the study include growing children and adolescents, BMI z-score is the most readily interpretable body composition parameter. Participants in the DCCR arm showed a reduction in BMI z-score, while participants in the placebo arm showed an increase in BMI z-score (LS mean difference [95% CI] −0.09 [−0.17, −0.01]). All domains of the FSZ suggested a tightening of environmental controls on food access in the placebo arm compared to the DCCR arm, consistent with the worsened hyperphagia in the placebo arm (Table 2). Other exploratory efficacy parameters favored DCCR compared to placebo but did not reach nominal significance (Table 2).

### Safety

No participant experienced an AE leading to study drug discontinuation. Similar proportions of participants in each arm experienced AEs (DCCR 73.7%, placebo 74.4%, Table 3). For participants experiencing a treatment-emergent AE, 93% experienced a grade 1 or grade 2 event. Seven (18.4%) participants in the DCCR arm and 11 (28.2%) participants in the placebo arm experienced 1 or more AEs related to study drug. The most common AEs related to DCCR were abnormal behavior (5.3%), affect lability (5.3%), hirsutism (5.3%), and hypertrichosis (5.3%), whereas the most common AEs related to placebo were hypertrichosis (12.8%), abnormal behavior (5.1%), and food craving (5.1%). One serious AE occurred in a participant in the placebo arm that was not considered related to the study drug (Table 3). Adverse events occurring in more than 10% of participants in either arm were nearly all common complications of PWS, and most occurred in the placebo arm at equivalent or higher rates than in the DCCR arm (Table 3). The most common AEs experienced by participants with PWS administered DCCR in DESTINY PWS and its long-term, open-label extension occurred infrequently in this study: increased hemoglobin A1c occurred in a single placebo participant while hyperglycemia occurred in a single DCCR participant; edema occurred in a single placebo participant, and hypertrichosis occurred in 2 DCCR participants and 5 placebo participants and in all instances was grade 1 (10, 11). As indicated, there was a single case of edema. The edema event was not characterized as either peripheral or pulmonary.

**Table 3.** Adverse events

Parameter	DCCR (n = 38) n (%)	Placebo (n = 39) n (%)	All participants (n = 77) n (%)
Number of participants who experienced at least 1 adverse event			
Adverse events	28 (73.7)	29 (74.4)	57 (74)
Adverse events related to study drug	7 (18.4)	11 (28.2)	18 (23.4)
Adverse events leading to premature discontinuation of study drug	0	0	0
Serious adverse event	0	1 (2.6)	1 (1.3)
Serious adverse events related to study drug	0	0	0
Adverse events occurring in ≥10% of participants in either arm			
Dermatillomania	5 (13.2)	6 (15.4)	11 (14.3)
Abnormal behaviors	5 (13.2)	5 (12.8)	10 (13.0)
Aggression	3 (7.9)	5 (12.8)	8 (10.4)
Hypertrichosis	2 (5.3)	5 (12.8)	7 (9.1)
Food craving	3 (7.9)	4 (10.3)	7 (9.1)
Anxiety	4 (10.5)	2 (5.1)	6 (7.8)
Hyperphagia*^a^*	4 (10.5)	1 (2.6)	5 (6.5)
Nasopharyngitis	1 (2.6)	4 (10.3)	5 (6.5)

Abbreviation: DCCR, diazoxide choline extended-release.

^*a*^Hyperphagia as an adverse event never occurred in isolation but always occurred in combination with other behavioral complications, and no occurrence of hyperphagia was determined by the investigator to be related to study medication. Hyperphagia as an adverse event was determined by the investigator whereas hyperphagia as the primary endpoint was assessed at every study visit using a caregiver-completed questionnaire.

There were small reductions in hemoglobin A1c over 16 weeks in both arms, with changes (SD) of −0.01 (4.054) mmol/mol in the DCCR arm and −1.29 (3.570) mmol/mol in the placebo arm from mean values of 38.11 and 36.53, respectively. One subject in each cohort received a new glucose-lowering medication. Empagliflozin was used in a DCCR-treated participant and semaglutide in a placebo-treated participant.

## Discussion

PWS is a complex, genetic neurobehavioral/metabolic syndrome that severely impacts patients, their caregivers, and their families. Hyperphagia is the highest priority unmet need in PWS not only because it has proven to be a very difficult aspect of the syndrome to manage but because it contributes to substantively lowered quality of life and increased mortality (5). Prior to the recent DCCR approval for the treatment of hyperphagia in adults and pediatric patients 4 years of age and older with PWS by the US Food and Drug Administration, no therapy has been approved, with failures of several drugs in phase 2 or phase 3 trials (20-23). This study is the first to demonstrate a therapeutic benefit that safely and effectively improves hyperphagia in people with PWS. Long-term treatment with DCCR improved hyperphagia against baseline and as compared to the natural history of the syndrome (11, 16). In this study, we established that hyperphagia worsens significantly when treatment with DCCR is withdrawn compared to continued DCCR administration. The LS mean increase in HQ-CT total score from baseline to 16 weeks in the placebo arm was 7.6 points from a baseline score of 8.1, representing a 93.8% increase from the RWP baseline. Given that a clinically meaningful worsening in HQ-CT score was an increase of 5 points from baseline, the change in HQ-CT score in placebo-treated patients was, on average, clinically meaningful. The results show that placebo-treated subjects increased the amount of time they asked or talked about food, stole or bargained for food more frequently, were more persistent in asking for food, and became more upset when denied food or stopped from asking about food, and food-related behaviors interfered more with their daily lives than did those on DCCR. Of note, the effect of DCCR withdrawal was gradual and became significant only at 12 weeks. The effect of withdrawal on HQ-CT total score appeared to be progressive and had not plateaued at 16 weeks. These observations are consistent with results from the prior open-label extension period where DCCR continued to reduce hyperphagia as measured by HQ-CT total score up until at least 39 weeks after initiating administration before plateauing (11). These results also suggest that there is no need to taper DCCR when it is discontinued.

BMI z-score moved in opposite directions in the 2 arms, rising in the placebo arm and dropping in the DCCR arm. These objective data directionally support the results for caregiver-assessed end results seen on the HQ-CT total score and PWSP domain scores. Every efficacy parameter, including the primary endpoint, the secondary endpoints, all domains of the PWSP questionnaire, and objective data including BMI z-score and weight favored the DCCR arm over the placebo arm, with a robust statistically significant difference between arms in the primary endpoint (*P* = .0022).

Based on the FSZ questionnaire, there was an obvious shift toward tightening of environmental controls on access to food in the placebo arm. This was most likely due to the increase in hyperphagia motivating caregivers to tighten controls.

DCCR was well tolerated, with similar rates of AEs in the 2 arms. There was a lower rate of AEs related to the study drug in the DCCR arm compared to placebo. There were no discontinuations of the study drug due to an AE and no serious AE in the DCCR arm. AEs reported in this study were also common complications of PWS and occurred at a similar or higher rate in the placebo arm compared to the DCCR arm. The most common AEs in the other phase 3 studies of DCCR in PWS (peripheral edema, hyperglycemia, and hypertrichosis) were reported infrequently in the RWP (10, 11).

There were some limitations of this study. First, there were 77 participants covering a wide age range and durations of drug exposure. The oldest participant was 29 years of age. While relatively young, this is above the median age and near the mean age at death in the PWS population. Although this is a modest population size that did not include individuals 30 years or older, baseline and demographic characteristics of these 77 patients compared to the initial 127 who were randomized in DESTINY PWS were similar (10). The study was appropriately designed and powered to detect differences in the primary endpoint. Therefore, we believe the findings are generalizable to the population of people with PWS. Second, the study was not powered to detect differences in subgroups, and therefore, firm conclusions cannot be drawn from subgroup analyses (Fig. S2) (19). Finally, there could be a concern that unblinding of treatment assignment due to treatment-emergent AEs, which might introduce bias and effect the results of the study. However, we do not believe this concern is valid. Participants in DCCR and placebo arms experienced similar AEs as those seen in the other phase 3 studies of DCCR in PWS: peripheral edema, hyperglycemia, and hypertrichosis. Of these, only hypertrichosis was reported in this study in at least 10% of participants in either arm and occurred somewhat more frequently in the placebo arm.

This study demonstrates significant and durable efficacy of a new drug, DCCR, for the treatment of hyperphagia in patients with PWS. The safety profile of DCCR was consistent with that seen in earlier studies and with common complications of PWS (10, 11). Data from this study as well as earlier studies together characterize the favorable, overall, long-term, benefit-risk profile of DCCR in patients with PWS.

## Data Availability

Data are available from the corresponding author on reasonable request.

## Clinical Trial Information

ClinicalTrials.gov number: NCT03714373.
